# Depressive Symptom Dimensions and Their Association with Hippocampal and Entorhinal Cortex Volumes in Community Dwelling Older Adults

**DOI:** 10.3389/fnagi.2018.00040

**Published:** 2018-02-20

**Authors:** Deirdre M. O’Shea, Vonetta M. Dotson, Adam J. Woods, Eric C. Porges, John B. Williamson, Andrew O’Shea, Ronald Cohen

**Affiliations:** ^1^Department of Aging and Geriatric Research, Center for Cognitive Aging and Memory, McKnight Brain Institute, University of Florida, Gainesville, FL, United States; ^2^Department of Clinical and Health Psychology, University of Florida, Gainesville, FL, United States; ^3^Department of Neuroscience, University of Florida, Gainesville, FL, United States; ^4^Department of Psychology, Georgia State University, Atlanta, GA, United States; ^5^Brain Rehabilitation Research Center, Malcom Randall Veterans Affairs Medical Center, Gainesville, FL, United States

**Keywords:** depressive symptom dimensions, hippocampal volumes, entorhinal cortex volumes, aging, older adults

## Abstract

**Objective:** Research has shown that depression is a risk factor for Alzheimer’s disease (AD) and subsequent cognitive decline. This is compounded by evidence showing an association between depression and reduced hippocampal volumes; a primary structure implicated in the pathogenesis of the disease. Less is known about the relationship between depression and other AD vulnerable regions such as the entorhinal cortex. Given the heterogeneity of depressive symptom presentation, we examined whether symptom dimensions were associated with hippocampal and entorhinal cortex volumes in community dwelling older adults.

**Methods:** Eighty-one community dwelling adults completed the Beck Depression Inventory – second edition and underwent structural neuroimaging. Measures of hippocampal and entorhinal cortex volumes were obtained using FreeSurfer software. Linear regression models included regions of interest as dependent variables, with depressive symptom dimensions, as independent variables, controlling for total intracranial volumes, age, education, and gender.

**Results:** Somatic symptoms were negatively associated with total, right, and left hippocampal volumes. Affective symptoms were negatively associated with total entorhinal cortex volumes, with a marginal main effect on left entorhinal cortex volumes.

**Conclusion:** Our findings provide support for examining depressive symptoms and their association with AD vulnerable regions along subdimensions of affective, cognitive, and somatic symptoms to better understand profiles of symptoms most associated with these regions. Conceptualizing depressive symptoms in this way may also better inform treatment approaches in terms of targeting types of symptoms that may be more closely linked to poorer brain and cognitive health outcomes.

## Introduction

Understanding the neurobiological basis of depression in late life has received considerable attention (for review, see Disabato and Sheline, 2012), in part due to the evidence that depression may be a risk factor or prodrome for incident dementia (Green et al., 2003; Brommelhoff et al., 2009). Several brain structures have been shown to be associated with depression including the prefrontal cortex, cerebellum, and the cingulate gyrus (Ito, 1986; Dotson et al., 2009; Koenigs and Grafman, 2009). However, the hippocampus has perhaps been the most well-studied structure in the context of major depressive disorder (MDD) (for reviews, see Koolschijn et al., 2009; Lorenzetti et al., 2009; McKinnon et al., 2009). Several studies have shown that MDD in older adults is associated with decreased hippocampal volume (Hickie et al., 2005; Ballmaier et al., 2008; Zhao et al., 2008). These findings are consistent with some behavioral studies that have shown an association between depression and reduced performance on episodic and declarative memory tests (Bäckman and Forsell, 1994; Airaksinen et al., 2007; Herrmann et al., 2007); abilities that are largely dependent on hippocampal integrity (Tulving and Markowitsch, 1998). However, the findings on this topic are mixed. Some studies have found bilateral hippocampal volume reduction (Sheline et al., 1996; MacQueen et al., 2003), while others have noted a differential impact of depression on the right (Steffens et al., 2000; Bell-McGinty et al., 2002) and left hippocampi (Bremner et al., 2000), and yet others have not found any association with hippocampal volumes or differences between hemispheres (Pantel et al., 1997; Vakili et al., 2000; Posener et al., 2003).

Research on subclinical depressive symptoms and their association with memory and the hippocampus has shown a similar pattern of mixed findings (Dotson et al., 2009; Goveas et al., 2011; Enache et al., 2015; O’shea et al., 2015; Zimmerman et al., 2016), suggesting that there may be similar underlying mechanisms, although this is less clearly understood.

The role for depression in preclinical Alzheimer’s disease (AD) is compounded by emerging research which has shown that depression in late life may be a risk factor or prodromal manifestation of an underlying disease pathology (Jorm, 2000; Ownby et al., 2006; Sun et al., 2008). Although the directionality of the association between depression and AD is yet to be established, some longitudinal research does suggest that depressive symptoms precede cognitive decline (Cui et al., 2007; Zahodne et al., 2014). If depressive symptoms are a risk factor or are related to AD vulnerable regions such as the hippocampus, then examining other AD vulnerable or related regions may further identify those who may be at an increased risk-most notably; the entorhinal cortex, a region closely associated both structurally and functionally with the hippocampus. Layer II of the entorhinal cortex provides excitatory input to the hippocampus via the performant pathway and layer IV of the entorhinal cortex receives one of the major efferent projections from the hippocampus. The entorhinal cortex can be thought of as a bridge linking the hippocampus to the neocortex (Zola-Morgan et al., 1994). It is also one of the first regions to show neuronal loss in preclinical AD and is severely affected in later stages of the disease process (Gomez et al., 2014). However, few studies have examined the association of depression on both the hippocampus and the entorhinal cortex in a single study. One study found that early onset depression was associated with hippocampal volumes but not entorhinal cortex volumes and the converse with late onset depression (LoD) such that LoD was associated with smaller entorhinal cortex volumes but not hippocampal volumes (Gerritsen et al., 2011). These findings suggest that there may be differential mechanisms underlying presentation of depression. Research from the pediatric literature also suggests that there is a differential association with brain regions in unipolar versus bipolar depressive disorders (Serafini et al., 2014) highlighting the significance of how depressive symptoms present and the way this alters brain integrity. However, whether these findings would translate to subclinical levels of depressive symptoms is not clear. In a more recent study, Gatchel et al. (2017) found that depressive symptoms were marginally (partial *r* = 0.183, *p* = 0.055) associated with cerebral tau 18F T807 levels in the entorhinal cortex and significantly associated with inferior temporal lobes (partial *r* = 0.188, *p* = 0.050) in cognitive normal older adults, providing some preliminary support for an association between subclinical depression and the entorhinal cortex.

A consideration of not only severity of depression but also how we address differences in depressive symptom presentation warrants further consideration. The latter has received much less examination, which is surprising given that we understand depression to be a heterogeneous disorder. It may be possible that the presentation of depressive symptoms may differentially impact cognition and brain structure. Factor analytic studies of self-reported depression measures have typically identified a somatic, cognitive, and affective component, albeit in various combinations (Shafer, 2006; Elhai et al., 2012), supporting the consensus that depression is a highly heterogeneous disorder (Winokur, 1997). Examining subdimensions of depression scales may better inform our understanding of the mechanisms underlying the relationship between depression and the brain that may be missed when using only whole scale sum scores (Fried et al., 2014; Fried and Nesse, 2015; Dotson, 2017).

Little has been reported in terms of depression symptom dimensions and two primary AD vulnerable regions; the hippocampus and the entorhinal cortex, despite the accumulating evidence that total depressive symptom severity is associated with hippocampal volumes and that depression may be a risk factor for AD. The present study’s overarching aim is to explore this potential relationship and examine whether subdimensions of depressive symptoms are differentially related to hippocampal volumes. A second aim of the study is to examine whether subclinical depressive symptoms and subdimensions are associated with a closely related structure; the entorhinal cortex. As noted earlier, much less is known about the association between depression and the entorhinal cortex relative to the hippocampus. Thus, in the present study, we hypothesized that greater total depressive symptoms would be associated with smaller hippocampal and entorhinal cortex volumes. Furthermore, we hypothesized that subdimensions of depressive symptoms would be differentially associated with these two brain regions.

## Materials and Methods

### Participants

Data from 81 older adults (59% female, mean age: 71; range: 43–93) were included in the present study. The study procedures were approved by the University of Florida Institutional Review Board. Participants were recruited from the north-central Florida community via newspaper advertising and fliers. All participants provided written and verbal informed consent prior to taking part in the study. Participants were excluded if they reported a diagnosis of any neurological or psychiatric disorders, suicidal ideation and intention (participants were further screened to assess whether there was concern for this, no participants fulfilled this criteria), substance abuse, as well as neurodegenerative disease such as Parkinson’s disease or AD or self-reported difficulties or concern with thinking or memory. Further exclusionary criteria were based on whether the participants had any magnetic resonance imaging (MRI) [3.0 Tesla (3T)] contraindications, including metal or medical devices inside the body, pregnancy, or claustrophobia. Sample characteristics are displayed in **Table 1**.

**Table 1:** Characteristics of the study sample (*n* = 81).

	Mean	*SD*
Age (years)	70.98	10.17
Sex (% female)	59.8	–
Education (years)	16.52	2.68
ICV	1,456,601.26	241,538.12
BDI-II total score	4.07	5.11
BDI-II affective subscale score	0.37	1.00
BDI-II cognitive subscale score	0.96	2.06
BDI-II somatic subscale score	2.73	2.83
Hippocampal volumes	–	–
Total	7,629.79	979.63
Right (mm)	3,886.15	496.86
Left (mm)	3,743.63	521.36
Entorhinal cortex volumes		
Total	3,237.87	519.61
Right (mm^2^)	1,510.25	309.12
Left (mm^2^)	1,727.63	301.26

*ICV, intracranial volume; BDI-II, Beck Depression Inventory – Second Edition*.

### Neuroimaging Procedure

Magnetic resonance imaging was performed using a 3T scanner (Achieva; Philips Electronics, Amsterdam, Netherlands) at the McKnight Brain Institute, University of Florida (Gainesville, FL, United States) with a 32-channel receive-only head coil. A high-resolution 3D T1 weighted MPRAGE scan was performed. Measures of hippocampal, entorhinal cortex, and intracranial volumes (ICV) were obtained using the following scanning parameters: voxel size = 1 mm isotropic; 1 mm slice thickness; TE = 3.2 ms; TR = 7.0 ms; FOV = 240 × 240; number of slices = 170; acquired in a sagittal orientation.

Magnetic resonance imaging T1-weighted scans were processed using FreeSurfer software (version 5.3^1^). Hippocampal and entorhinal cortex volumes were obtained from the automatic subcortical segmentation stream. This method has shown to be reliable and produce accurate results of subcortical regions (Fischl et al., 2002; Jovicich et al., 2009). Each slice of all scans acquired was manually inspected by trained technicians and any errors in segmentation were manually corrected and re-processed through FreeSurfer. Total hippocampal and entorhinal cortex volumes were computed by summing the right and left volumes.

### Depressive Symptoms

Depressive symptoms were measured using the Beck Depression Inventory – Second Edition (BDI-II; Beck et al., 1996). This is a widely used 21-item questionnaire that is used to assess for the presence of depressive symptoms over the past 2 weeks. Each item has a score between 0 and 3, thus total scores can range from 0 to 63. The range in the present sample was between 0 and 13, which indicates subclinical level of symptoms. The BDI-II has been previously shown to have strong psychometric properties in community dwelling older adults (Segal et al., 2008). The BDI-II has also been shown to have a three-factor structure reflecting a cognitive, somatic, and affective dimension that is present in both clinical and non-clinical samples (Vanheule et al., 2008). **Table 2** shows each item corresponding to each of the three subscales used in the present study.

**Table 2:** Item content of the BDI-II subscales.

Affective symptoms	Cognitive symptoms	Somatic symptoms
Sadness	Past failure	Crying
Pessimism	Feelings of guilt	Agitation
Loss of pleasure	Feelings of punishment	Loss of energy
Loss of interest	Dis-like of self	Changes in sleeping pattern
Suicidal thoughts or wishes	Self-criticism	Irritability
	Indecisiveness	Changes in appetite
	Worthlessness	Concentration difficulty
		Tiredness or fatigue
		Loss of interest in sex

*BDI-II, Beck Depression Inventory – Second Edition*.

### Statistical Analyses

All analyses were performed using SPSS version 22 (IBM Corp., Armonk, NY, United States). Data were screened for outliers by computing standardized *z*-scores. Outliers were identified as *z* > ±3 standard deviations above the mean and removed from analyses (*n* = 5). Several linear regression analyses were performed to examine the independent associations between total BDI-II scores on total hippocampal and entorhinal cortex volumes, controlling for covariates. BDI-II subdimensions were regressed onto the following outcome measures; total, right, and left hippocampal volumes and total right and left entorhinal cortex volumes. Age (continuous), sex, education (continuous), and ICV (continuous) were used as covariates in all the models. Covariates were entered in the first step of each model, followed by the cognitive, affective, and somatic symptom in steps 2, 3, and 4 (respectively) so that the effect of each of the scales could be estimated. All *z*-scored variables were used to facilitate interpretation of effects. Alpha was set at ≤0.05.

## Results

Results from the linear regression analyses revealed that older age was negatively associated with total, right, and left hippocampal volumes (**Table 3**). Increased age was also negatively associated with left entorhinal cortex volumes. Sex and education were not significant independent predictors in any of the models.

**Table 3:** Results of the regression analyses.

	Model 1 B (Se)	Model 2 B (Se)	Model 3 B (Se)	Model 4 B (Se)	Model 5 B (Se)	Model 6 B (Se)
	
Final	Thippo	Tentor	Lhippo	Rhippo	Lentor	Rentor
Age	-0.693 (0.154)**^∗∗∗^**	-0.432 (0.178)**^∗∗^**	-0.621 (0.164)**^∗∗∗^**	-0.720 (0.151)**^∗∗∗^**	-0.381(0.182)**^∗^**	-0.348 (0.198)
Sex	-0.031 (0.118)	-0.006 (0.137)	-0.001 (0.126)	-0.060 (0.116)	0.061 (0.140)	-0.010 (0.145)
Education	-0.094 (0.113)	0.122 (0.131)	-0.038 (0.121)	-0.146 (0.111)	0.196 (0.134)	0.010 (0.145)
ICV	0.093 (0.124)	0.405 (0.143)**^∗∗^**	0.029 (0.130)	0.154 (0.121)	0.293 (0.146)**^∗^**	0.390 (0.159)**^∗^**
Cognitive	0.051 (0.108)	0.026 (0.142)	0.024 (0.130)	0.077 (0.120)	0.076 (0.145)	0.120 (0.157)
Affect	-0.046 (0.146)	-0.382 (0.169)**^∗^**	-0.115 (0.156)	0.046 (0.130)	-0.336 (0.175)	-0.209 (0.188)
Somatic	-0.411 (0.136)**^∗∗^**	-0.023 (0.171)	-0.326 (0.157)**^∗^**	-0.471 (0.134)**^∗∗^**	-0.027 (0.174)	-0.011 (0.189)
***R*^2^ change**		
Step 1	0.358	0.274	0.288	0.389	0.211	0.207
Step 2 *R*^2^	0.013	0.003	0.015	0.010	0.019	0.001
Step 3 *R*^2^	0.024	0.069	0.032	0.014	0.057	0.041
Step 4 *R*^2^	0.068	0.000	0.043	0.086	0.000	0.000

*All variables are in *z*-score metric. Thippoca, total hippocampal volumes; Tentor, total entorhinal cortex volumes; Rhippo, right hippocampal volumes; Rhippo, left hippocampal volumes; Rentor, right entorhinal cortex volumes; Lentor, left entorhinal cortex volumes; ICV, intracranial volume; Cognitive, cognitive subscale of the Beck Depression Inventory – Second edition; Affect, affective subscale of the Beck Depression Inventory – Second edition; Somatic, somatic subscale of the Beck Depression Inventory – Second edition*.

### Total Symptoms

There was a significant main effect of total depressive symptoms on total hippocampal volumes but not total entorhinal cortex volumes. Greater depressive symptoms were associated with a decrease in total hippocampal volumes.

### Symptom Dimensions

#### Cognitive

There was no main effect of cognitive symptoms on any of the outcome variables.

#### Affective

Higher scores on the affective symptom dimension were negatively associated with total entorhinal cortex volumes and marginally with left entorhinal cortex volumes (*p* = 0.056). Affective symptoms accounted for an additional 7% of the variance in total entorhinal cortex volumes, and almost 6% additional variance in left entorhinal cortex volume.

#### Somatic

Higher somatic depressive symptoms were negatively associated with total, right, and left hippocampal volumes but there was not a statistically significant association between this domain and entorhinal cortex volumes. The addition of somatic symptoms accounted for almost 7% of the variance in total hippocampal volumes, and 4% and almost 9% of the variance in left and right hippocampal volumes, respectively.

## Discussion

We found that total depressive symptoms were negatively associated with total hippocampal volumes but not entorhinal cortex volumes. The association between total depressive symptoms and total hippocampal volumes was likely driven by somatic symptoms as this was the only subdimension significantly associated with total, left, and right hippocampal volumes. There was no association between somatic symptoms and entorhinal cortex volumes. Additionally, there was a significant negative association between affective symptoms and total entorhinal cortex volumes. There was also a marginally significant association between affective symptoms and left entorhinal cortex volumes. There were no other association between depressive symptom dimensions and any of the other brain regions. Our investigation of the subdimensions of depressive symptoms showed that somatic symptoms were the only symptom domain associated with hippocampal volumes. This is consistent with previous work which showed that somatic symptoms, but not affective or cognitive, were associated with thinner cortical precuneus volumes in older adults (Szymkowicz et al., 2017) as well as reduced overall gray and white matter volumes (Dotson et al., 2013; Kirton et al., 2014; Tang et al., 2014; Thames et al., 2015). Together, these findings highlight the significance of examining depressive symptoms in this way. Using only total scores may mask specific effects of groups of items or dimensions of depressive symptoms on brain volumes.

One explanation for the association between somatic symptoms and hippocampal volumes may be that some of the items underlying this dimension (“loss of energy,” “changes in sleeping patterns,” “irritability,” “tiredness or fatigue,” “concentration difficulties”) may reflect sleep quality. Previous research has shown a link between sleep quality/duration and brain volumes (Chao et al., 2014; Bernardi et al., 2016), including hippocampal volumes in older adults (Elcombe et al., 2015) and hippocampal glial integrity (Cross et al., 2013), although this topic in older adults is underexplored. The relationship between depression and sleep is well established (for reviews, see Tsuno et al., 2005; Bao et al., 2017), but the mechanisms through which these are related remain unclear. Disruptions in sleep quality and efficiency have been explained in terms of circadian rhythms disruptions due to abnormalities in plasma melatonin levels. Low plasma melatonin production at night is related to disruptions in circadian rhythms (Dawson and Encel, 1993).

Depression, as has largely been explained in terms of excess glucocorticoid production which proposes that depression is a type of stress response involving a chronic production of glucocorticoids which implicate the hypothalamic, pituitary, and limbic systems (Sheline, 2011). Several studies have shown that cumulative exposure to glucocorticoids has been related to hippocampal (i.e., a core limbic structure) volume reduction and impairments in hippocampal-dependent cognitive tasks in rodents (Woolley et al., 1990; Watanabe et al., 1992; Lee et al., 2017; Nguyen et al., 2018). Studies in humans have shown similar findings in healthy older adults who report high levels of stress concurrent with high levels of cumulative cortisol levels (Newcomer et al., 1999; Gold, 2015; Radley et al., 2015). There is also evidence that reductions in brain-derived neurotrophic factor (BDNF) following prolonged stress is associated with decreased hippocampal volumes (Mondelli et al., 2011). Excess glucocorticoid production is proposed to increase neuronal vulnerability to aging effects and/or pathological processes (Bishop et al., 2010).

Several studies have shown, for example, that about 50% of those with depression also have HPA hyperactivity (Arana et al., 1985; Checkley, 1996; Arborelius et al., 1999). Some research suggests that depression and sleep disorders may share some pathophysiological features (Thase, 2006; Srinivasan et al., 2009). This is supported by evidence which showed that the release of corticotropin-releasing hormone (CRH) in response to stress inhibits melatonin production in depressed patients (Kellner et al., 1997). However, further research is required to draw any firm conclusions.

Interpreting the relationship between affective symptoms of depression and entorhinal cortex volumes is more difficult to explain, largely due to the paucity of research on this topic. The differential impact of symptom subdimensions on the hippocampus versus entorhinal cortex may mirror findings which have shown that early-onset depression is association with hippocampal volumes reduction, whereas LoD is associated with entorhinal cortex volume reduction (Gerritsen et al., 2011). We may speculate that chronic stress due to life time or early experiences with depression impacts the hippocampus via pathophysiological processes outlined above and are more related to somatic symptoms. While the association between affective symptoms and entorhinal cortex (one of the first regions to be implicated in AD) may reflect an affective reaction to overt problems (such as memory lapses) due to an underlying preclinical AD process. However, we did not have information about time of onset of depressive symptoms to explore this further, which is a limitation to explaining our findings. Additionally, longitudinal studies would be better suited to explore this possibility and determine whether there is a differential relationship between subdimensions of depression and incidence of AD.

An alternative perspective might consider the role of neuroinflammation and its relation to depressive symptoms. Accumulating evidence suggest that chronic and low grade neuroinflammation may contribute to the pathogenesis of stroke, depression, and dementia as it alters molecular and cell function (for review, see Uzoni et al., 2015). Studies have shown that in MDD there are higher rates of neuroinflammation than in non-depressed participants (Tiemeier et al., 2003) and that elevated cytokines (inflammatory markers) can alter hippocampal neurogenesis (Maes et al., 2009; Caraci et al., 2010). Although less is known regarding the association between subclinical depressive symptoms and pro-inflammatory markers, some studies have shown that elevated cytokines IL-6 and Il-8 are associated with depressive symptoms (Penninx et al., 2003; Krishnadas and Cavanagh, 2012). Although the mechanisms underlying depression in the elderly are poorly understood, targeting and treating pro-inflammatory risk factors such diabetes and hypertension may optimize brain health by mitigating the effect of inflammation on molecular processes contributing to poor brain health and emergence of depressive symptoms. Other studies have also shown that exercise reduces levels of inflammation and depressive symptoms (Cotman et al., 2007; Trivedi et al., 2011), which may also be a viable treatment or protective lifestyle factor.

Additionally, with regard to brain health and protective mechanisms in the context of treatment, increasing brain or cognitive reserve may modulate responses to stress both at the molecular and psychological levels. Indeed, a more engaged and active lifestyle (a proxy of cognitive reserve) may protect against depressive symptoms (Van Gool et al., 2007). Additionally, other studies have shown that in healthy elderly and mild cognitive impairment samples with high cognitive reserve, there was greater network connectivity in the left frontal cortex (Franzmeier et al., 2017a,b), a region which supports memory functioning. Thus, the impact of depressive symptoms on brain integrity may be off-set by increasing cognitive reserve via engaging in a more stimulating and active lifestyle. Potentially, depressive symptoms could be treated from a multi-modal approach, one which focuses on enhancing brain health.

There are other limitations to our study that should be noted. The cross-sectional design is a limitation insofar as elucidating the causal mechanisms underlying the associations between symptom dimensions and hippocampal and entorhinal cortex volumes. For example, do somatic and affective symptoms cause hippocampal and entorhinal cortex volume reductions, respectively, or do smaller hippocampal and entorhinal cortex volumes cause one to be more susceptible to these symptoms? Follow-up studies will be required to better address this question. Another limitation includes the relatively small sample size and relatively homogenous Caucasian ethnicity of participants in the present study which may impact the generalizability of our findings. Future research should include a more ethnically diverse and larger sample size and/or examine whether these findings differ across race/ethnicity. Given that some previous work has shown that depressive symptom presentation in older adults vary as a function of ethnic status (Myers et al., 2002; Williams et al., 2015), this would seem important. Additionally, although we presented studies that support the hypothesis that depression may be a prodromal manifestation in AD, not all studies have provided consistent evidence (Rej et al., 2015; Almeida et al., 2016) and so caution should be exercised when considering that the presence of depressive symptoms may a prodrome of AD. Understanding the biological mechanisms underlying these two disorders will help bring clarity to this topic. Longitudinal studies will be necessary to fully understand this association. Another interesting avenue of research might examine whether depressive symptoms during a euthymic period also increases the risk for neurocognitive and brain dysfunction as this was not addressed in the present study.

## Conclusion

Our findings provide support for examining depressive symptoms and their association with AD vulnerable regions along subdimensions of affective, cognitive, and somatic symptoms to better understand profiles of symptoms most associated with these regions. This approach may help to parse out those who may be at an increased risk of AD or region-dependent cognitive decline. Conceptualizing depressive symptoms in this way may also better inform treatment approaches in terms of targeting types of symptoms that may be more closely linked to poorer brain and cognitive health outcomes. For example, more prevalent self-reported somatic symptoms may signal the need to address lifestyle factors such as sleep, as well as physical and cognitive activity levels, and management of cardiovascular risk factors (i.e., diabetes and hypertension), whereas greater reports of affective symptoms may signal cognitive dysfunction or accelerated cognitive decline. Future research addressing the limitations discussed above would help to confirm and generalized these findings.

## Ethics Statement

The study procedures and protocol were approved by the University of Florida Institutional Review Board. All participants provided written and verbal informed consent prior to taking part in the study in accordance with the Declaration of Helsinki.

## Author Contributions

DO was involved in all aspects of the manuscript including hypothesis formation, statistical analyses, and writing of the manuscript. AO conducted volumetric analyses that were used in the manuscript. EP, JW, AW, and RC were involved in the conception of the study and RC was involved in the conception of the manuscript. All authors contributed to the intellectual content and approved the final version to be published.

## Conflict of Interest Statement

The authors declare that the research was conducted in the absence of any commercial or financial relationships that could be construed as a potential conflict of interest.

## Footnotes

1 http://surfer.nmr.mgh.harvard.edu
